# *Viola tricolor* Induces Apoptosis in Cancer Cells and Exhibits Antiangiogenic Activity on Chicken Chorioallantoic Membrane

**DOI:** 10.1155/2014/625792

**Published:** 2014-08-28

**Authors:** Hamid Reza Sadeghnia, Taghi Ghorbani Hesari, Seyed Mohsen Mortazavian, Seyed Hadi Mousavi, Zahra Tayarani-Najaran, Ahmad Ghorbani

**Affiliations:** ^1^Neurocognitive Research Center, School of Medicine, Mashhad University of Medical Sciences, Mashhad 9177948564, Iran; ^2^Pharmacological Research Center of Medicinal Plants, School of Medicine, Mashhad University of Medical Sciences, Mashhad 9177948564, Iran; ^3^Department of Pharmacology, School of Medicine, Mashhad University of Medical Sciences, Mashhad 9177948564, Iran; ^4^Department of Pharmacodynamics and Toxicology, School of Pharmacy, Mashhad University of Medical Sciences, Mashhad 917751365, Iran

## Abstract

In the present study, the cytotoxic and apoptogenic properties of hydroalcoholic extract and ethyl acetate (EtOAc), *n*-butanol, and water fractions (0–800 *μ*g/mL) of* Viola tricolor* were investigated in Neuro2a mouse neuroblastoma and MCF-7 human breast cancer cells. In addition, antiangiogenic effect of EtOAc fraction was evaluated on chicken chorioallantoic membrane (CAM). The quality of EtOAc fraction was also characterized using high performance liquid chromatography (HPLC) fingerprint. Cytotoxicity assay revealed that EtOAc fraction was the most potent among all fractions with maximal effect on MCF-7 and minimal toxicity against normal murine fibroblast L929 cells. Apoptosis induction by EtOAc fraction was confirmed by increased sub-G1 peak of propidium iodide (PI) stained cells. This fraction triggered the apoptotic pathway by increased Bax/Bcl-2 ratio and cleaved caspase-3 level. Moreover, treatment with EtOAc fraction significantly decreased the diameter of vessels on CAM, while the number of newly formed blood vessels was not suppressed significantly. Analysis of quality of EtOAc fraction using HPLC fingerprint showed six major peaks with different retention times. The results of the present study suggest that* V. tricolor* has potential anticancer property by inducing apoptosis and inhibiting angiogenesis.

## 1. Introduction

Cancer is a devastating disease with tremendous negative implications at the personal, health care, economical, and social levels. It figures among the leading causes of death worldwide, accounting for 8.2 million deaths in 2012 [1].

Excluding skin cancers, breast cancer is the most common malignancy and the second leading cause of cancer death among women [2]. Breast cancer is a heterogeneous disease encompassing multiple subgroups with differing molecular signatures, prognoses, and responses to therapies. Although, current treatment options for breast cancer are moving toward nontoxic, potent targeted therapies that can be tailored to an individual patient's tumor, the development of resistance to all of these therapies is an ongoing challenge [3].

Neuroblastoma accounts for disproportionate morbidity and mortality among the cancers of childhood. It is a complex and heterogeneous disease and, despite recent advances, 50 to 60% of patients with high-risk neuroblastoma have a relapse, and to date there are no salvage treatment regimens known to be curative [4].

Phytochemicals from herbs are becoming increasingly important sources of anticancer drugs or compounds for cancer chemoprevention or adjuvant chemotherapy [5]. Recently, some chemopreventive extracts of herbs have been shown to be antitumorigenic [6, 7]. The anticancer effects have been shown to be mediated through inhibiting cancer-activating enzymes, enhancing DNA repair processes, immunomodulatory or antioxidant actions [8].

*Viola tricolor*, a member of Violaceae plant family, is common horticultural plant in Iran. It has been reported to have a number of medicinal attributes including anti-inflammatory [9], antimicrobial [10], antioxidant [11, 12], sedative [13], and diuretic [14] activities. Recent studies have shown that* Viola tricolor *contains cyclotide compounds with cytotoxic properties [15]. In our previous preliminary works, we have shown the cytotoxic activity of* V. tricolor *and its *n*-butanol or ethyl acetate fractions on neuroblastoma and uterine cervix carcinoma cells [16, 17], but the exact mechanistic pathways for this cytotoxicity were remained to be clear. In the present study, the cytotoxic and apoptogenic properties of* V. tricolor *in Neuro-2a mouse neuroblastoma and MCF-7 human breast cancer, as well as normal murine fibroblast L929 cells, were investigated. The possible inhibitory effect of* V. tricolor *on angiogenesis in chicken chorioallantoic membrane was also investigated. In addition, the quality of EtOAc fraction of* V. tricolor* was characterized by high performance liquid chromatography (HPLC) fingerprint.

## 2. Materials and Methods

### 2.1. Chemicals and Reagents

The 3-(4,5-dimethylthiazol-2-yl)-2,5-diphenyl tetrazolium (MTT), doxorubicin, dimethyl sulfoxide (DMSO), propidium iodide (PI), protease inhibitor cocktail, phosphatase inhibitor cocktail, sodium citrate, Triton X-100, phenylmethylsulfonyl fluoride (PMSF), and bicinchoninic acid (BCA) protein assay kit were purchased from Sigma (St. Louis, USA). Dulbecco's Modified Eagles Medium (DMEM) and fetal bovine serum (FBS) were bought from Gibco (Life technologies, Carlsbad, USA). Anti-*β*-actin, Bax, Bcl-2, and horseradish peroxidase- (HRP-) conjugated goat anti-rabbit IgG antibodies were obtained from Cell Signaling Technology (Danvers, USA). All the solvents used for extraction were also purchased from Caledon (Canada).

### 2.2. Preparation of* V. tricolor *Extract and Its Fractions

The* V. tricolor *aerial parts of the flowering plants were collected from Pardis Campus (Mashhad, northeast of Iran) and authenticated by the herbarium of School of Pharmacy (Mashhad University of Medical Sciences, Iran; voucher specimen number 12568). The plant materials were washed, dried, powdered, and subjected to extraction with 70% ethanol (EtOH/H_2_O 70 : 30) in a Soxhlet apparatus for 48 h. The hydroalcoholic extract (HAE) was then dried on a water bath (45°C, 2 h) and the yield (32%) was kept at −20°C until use.

For preparation of fractions, the dried hydroalcoholic extract (10 g) was suspended in distilled water and transferred to a separator funnel. With solvent-solvent extraction, it was fractionated using ethyl acetate (EtOAc) and *n*-butanol. The ethyl acetate and *n*-butanol fractions were then separated to obtain water (H_2_O) fraction [18, 19]. The solvents of the fractions were then evaporated and the residues were dissolved in phosphate buffered saline (PBS, pH 7.4) solution containing 0.5% DMSO (for EtOAc and *n*-butanol fractions) or PBS alone (for water fraction).

### 2.3. Cell Culture and Treatment

The MCF-7, Neuro2a, and normal L929 cells were cultivated in high-glucose DMEM supplemented with 10% FBS and penicillin (100 units/mL) and streptomycin (100 *μ*g/mL) at 37°C in an atmosphere of 5% CO_2_. Trypsin solution was used to passage cultures whenever they were grown to about 70% confluence. The cells at subconfluent stage were harvested from culture flask and, after checking the viability with trypan blue exclusion technique, they were seeded overnight in 96-well culture plate. Then, to test the possible cytotoxicity of* V. tricolor*, the culture medium was changed by fresh one containing varying concentrations (0–800 *μ*g/mL) of the HAE and its fractions. Then, the cells were further incubated for 24 h.

### 2.4. MTT Assay

The effect of* V. tricolor *on MCF-7, Neuro2a, and L929 cells proliferation was determined using MTT colorimetric assay as previously described [20, 21]. Briefly, at the end of treatment, the MTT solution was added to each well of culture plate and the reaction mixture was incubated for 2 h. Then, the mixture was removed and the resulting formazan dissolved in DMSO. The optical density of formazan dye was read at 570 and 620 nm (background) using a StatFAX303 plate reader. All experiments were carried out in triplicate.

### 2.5. PI Staining

MCF-7 and Neuro2a cells were seeded overnight in 12-well culture plate (75000 cells/well) and treated for 24 h with tested drugs. Then floating and adherent cells were harvested and incubated with 750 *μ*L of a hypotonic buffer (50 *μ*g/mL propidium iodide in 0.1% sodium citrate containing 0.1% Triton X-100) at 4°C overnight in the dark [22]. Samples were then analyzed with BD FACSCanto flow cytometer (BD Biosciences, San Jose, CA). A total of 10,000 events per sample were obtained and the data was analyzed using WinMDI (version 2.8) software. Three independent experiments were performed.

### 2.6. Western Blotting Analysis

After treatment, the Neuro2a cells were incubated with lysis buffer (50 mM Tris-Hcl, 150 mM NaCl, 2 mM EDTA, 5 mM sodium fluoride, 1 mM NaVO_4_, 1% Nonidet P-40, and protease and phosphatase inhibitors) and centrifuged and the protein concentration of the supernatants was measured using BCA kit. Equal amounts of protein from samples were mixed with loading buffer and boiled for 5 min. Samples were separated by electrophoresis, incubated in a blocking buffer (50 mM Tris/HCl, 150 mM NaCl, 0.1% Tween 20, and 5% skimmed milk) and the blots were probed with antibodies. The bound antibody was made visible using HRP-conjugated goat anti-rabbit secondary antibody and an enhanced chemiluminescence system. Bands were analyzed using Gel Pro Analyzer Software (Media Cybernetics) and normalized in respect to corresponding *β*-actin band and expressed as fold of control [23].

### 2.7. Chicken Chorioallantoic Membrane (CAM) Angiogenesis Model

Fertilized chicken eggs were incubated at 37°C and 70% relative humidity in a forced draught incubator. At day 8, a 1.5–2 cm window was opened aseptically on each egg shell, exposing the part of the CAM which contained the central vein and 20 or 40 *μ*g/egg of the ethyl acetate fraction was injected into the chorioallantoic sac. The control eggs received the same volume sterile PBS only. Then, the windows were sealed with sterile Parafilm and the eggs were then returned to the incubator. At day 12, the seals were removed and the CAM vasculatures were photographed using a stereo microscope equipped with a digital camera (Canon EOS 40D with Canon EF 100 mm f/2.8 USM lens). The angiogenic response was evaluated by counting the vessel density using ImageJ software [24].

### 2.8. Characterization of the EtOAc Fraction of* V. tricolor* by HPLC

The quality of EtOAc fraction of* V. tricolor* was characterized by HPLC-UV fingerprint [25]. The Waters HPLC (Waters Association, Milford, MA, USA) apparatus consisted of a model 510 Waters pump, a 20 *μ*L Rheodyne 7725 injector, and a variable wavelength model 486 Waters UV-VIS detector. The chromatograms were analyzed using Autochoro 3000 data module (Young Lin Instruments, South Korea). The chromatographic separation was carried out with a reverse-phase Waters C18 analytical column (250 × 4.6 mm, 5 *μ*m particle size). An isocratic elution was performed by the mobile phase of 20% acetonitrile and 80% phosphoric acid (0.085%, pH = 2.2) at a flow rate of 1 mL/min. The UV detector wavelength was set at 340 nm. A sample of the EtOAc fraction was dissolved in mobile phase and passed through 0.45 *μ*m membrane filter. Then, 20 *μ*L of sample (500 *μ*g/L) was injected into the HPLC column.

### 2.9. Statistical Analysis

All results are presented as mean ± standard error of the mean (SEM) of experiments performed in triplicate. The Kolmogorov-Smirnov test was first performed to assess the normality assumption of the data. The values were normally distributed and therefore compared using the one-way analysis of variance (ANOVA) followed by Tukey's post hoc test for multiple comparisons. The *P* values less than 0.05 were considered to be statistically significant.

## 3. Results

### 3.1. *V. tricolor* Extract and Its Fractions Induce Cell Death of MCF-7 and Neuro2a Cells

**Figure 1:**
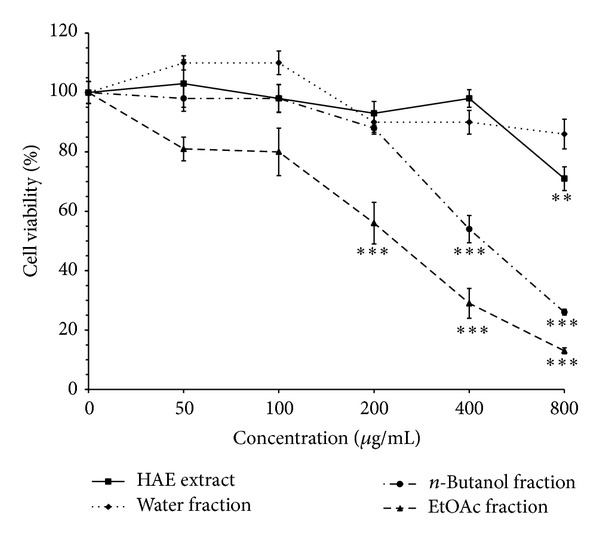
Effects of* Viola tricolor* on proliferation of MCF7 cells. The cells were treated with increasing concentrations of hydroalcoholic (HAE) extract or its water, *n*-butanol, or ethyl acetate (EtOAc) fractions for 24 h. The percent of viable cells was normalized against untreated control cells (0 *μ*g/mL). Data are mean ± SEM of three independent experiments performed in triplicate. **P < 0.01 and ***P < 0.001 versus untreated control cells.

**Figure 2:**
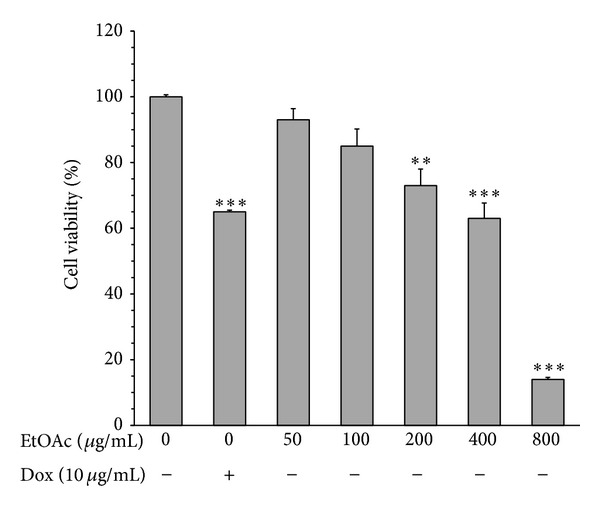
Effects of ethyl acetate (EtOAc) fraction of* Viola tricolor* on proliferation of Neuro2a cells. The cells were treated for 24 h and then percent of viable cells (quantified by MTT assay) was normalized against untreated control cells (0 *μ*g/mL). Data are mean ± SEM of three independent experiments performed in triplicate. **P < 0.01 and ***P < 0.001 versus untreated control cells.

**Figure 3:**
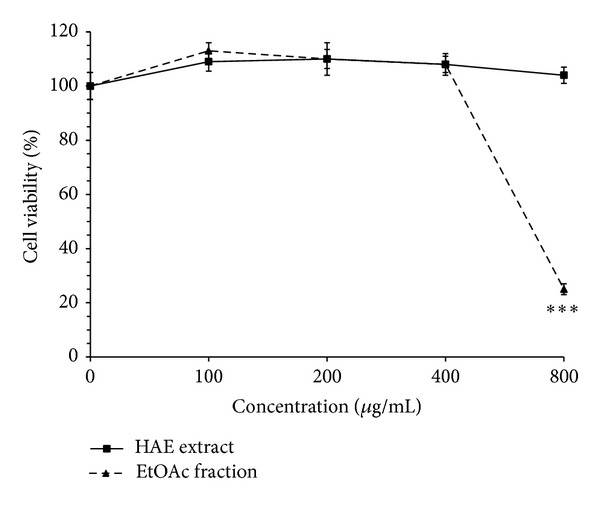
Effects of* Viola tricolor* hydroalcoholic (HAE) extract and its ethyl acetate (EtOAc) fractions on proliferation of L929 cells. The cells were treated for 24 h and then percent of viable cells (quantified by MTT assay) was normalized against untreated control cells (0 *μ*g/mL). Data are mean ± SEM of three independent experiments performed in triplicate. ***P < 0.001 versus untreated control cells.

Hydroalcoholic extract (HAE) of* V. tricolor *and EtOAc, *n*-butanol, and H_2_O fractions were examined for cytotoxic potential on MCF-7, Neuro2a, and normal cells (L929). Cells were incubated with increasing concentrations of the extract and its different fractions (0–800 *μ*g/mL) for 24 h. Results demonstrated that the extract decreased cell viability in a concentration-dependent manner (Figures 1, 2, and 3). As shown in Figure 1, the cytotoxic potential of HAE was seen only at high concentration (800 *μ*g/mL, *P* < 0.01); on the other hand, cell survival was not significantly affected by treatment of MCF-7 cells with water fraction at all concentrations tested (*P* > 0.05). Among the fractions, EtOAc fraction showed the most cytotoxic effects on cancer cells, but limited toxicity on normal cells (Figures 1, 2, and 3). Significant inhibition (about 50%) of cell proliferation by EtOAc was seen at concentration of 200 *μ*g/mL (*P* < 0.001) in MCF-7 cells (Figure 1). Compared to Neuro2a cells, MCF-7 cells were found to be more sensitive to cytotoxic effects of the EtOAc fraction. As illustrated in Figure 2, although signs of toxicity in Neuro2a cells (about 25% inhibition in cell proliferation) were observed at concentration of 200 *μ*g/mL (*P* < 0.01), the greatest effect appeared only at higher concentrations (800 *μ*g/mL, *P* < 0.001). As shown in Figure 3, cell growth and survival were not significantly affected by treatment of L929 with concentrations up to 400 *μ*g/mL of HAE and its EtOAc fraction (*P* > 0.05, after 24 h of treatment). The data also indicated that treatment of Neuro2a cells with doxorubicin (10 *μ*g/mL) for 24 h resulted in significant inhibition of cell viability, as compared with control (*P* < 0.001) (Figure 2).

### 3.2. EtOAc Fraction of* V. tricolor* Induces Apoptosis of MCF-7 and Neuro2a Cells

**Figure 4:**
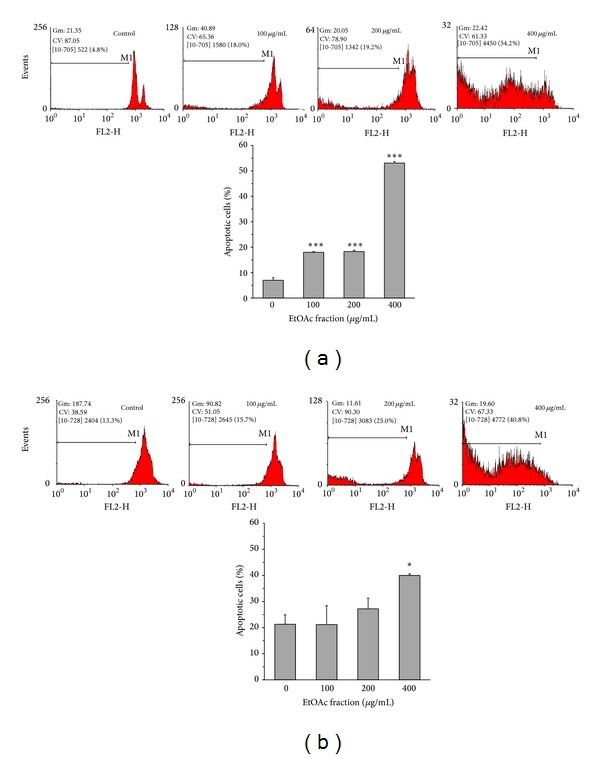
Effects of ethyl acetate (EtOAc) fraction of* Viola tricolor* on apoptosis of MCF7 (a) and Neuro2a (b) cells. The cells were treated for 24 h and then incubated with a hypotonic buffer containing propidium iodide and Triton X-100. Then, the cells were analyzed with a flow cytometer. Data are mean ± SEM of three independent experiments performed in triplicate. *P < 0.05 and ***P < 0.001 versus untreated cells (0 *μ*g/mL).

To determine if apoptosis is involved in the cytotoxic effects of EtOAc fraction of* V. tricolor*, apoptotic cells were determined by PI staining of DNA fragments by flow cytometry (sub-G1 peak). Cells were exposed to various concentrations (0, 100, 200, and 400 *μ*g/mL) of EtOAc fraction for 24 h.* V. tricolor* treatment of the cancer cell lines significantly increased the sub-G1 peak with a concomitant decrease in G1 phase, compared to the untreated control cells (Figures 4(a) and 4(b)).

### 3.3. EtOAc Fraction of* V. tricolor* Increases the Cleaved Caspase-3 and Bax/Bcl-2 Ratio

**Figure 5:**
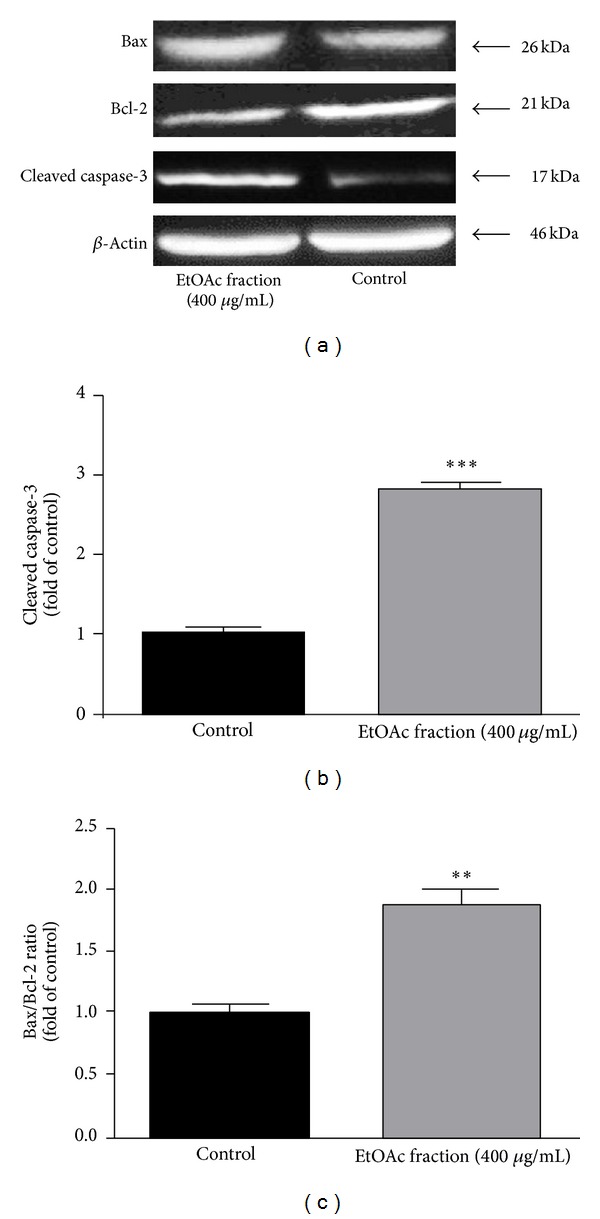
Effects of ethyl acetate (EtOAc) fraction of* Viola tricolor* on proapoptotic (Bax, caspase-3) and antiapoptotic (Bcl-2) proteins expression in Neuro2a cells (a). The cell lysates were immunoprecipitated with anti-Bax, anti-Bcl-2 and anti-cleaved caspase-3 antibodies and then immunoblotted using horse radish peroxidase-conjugated goat anti-rabbit secondary antibody with *β*-actin as a protein loading control. Data are presented as the fold induction over control cells ((b)-(c)). **P < 0.01, ***P < 0.001 versus untreated control cells.

The increase in the active form of caspase 3 and Bax/Bcl-2 ratio were used as an indicator of apoptosis. B-cell lymphoma-2 (Bcl-2) and Bcl-2 associated X protein (Bax), members of the Bcl-2 family of proteins, are antiapoptotic and proapoptotic factors, respectively. The ratio of Bax/Bcl-2 proteins plays a pivotal role in controlling cytochrome c release and apoptosis initiation via the mitochondrial (intrinsic) pathway [26]. Our results indicated that EtOAc fraction of* V. tricolor *(400 *μ*g/mL) acts by downregulating Bcl-2 and upregulating Bax protein expression in Neuro2a cells (Figures 5(a) and 5(c)).

In addition, enhanced caspase-3 level following treatment of Neuro2a cells with EtOAc fraction of* V. tricolor *(400 *μ*g/mL) was seen, indicating caspase-dependent apoptosis (Figure 5(b)).

### 3.4. EtOAc Fraction of* V. tricolor* Inhibits CAM Angiogenesis

**Figure 6:**
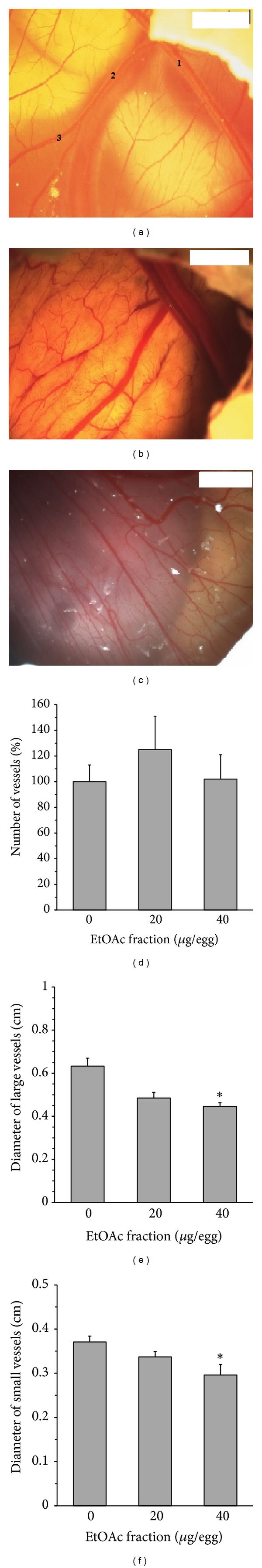
Effect of ethyl acetate (EtOAc) fraction of* V. tricolor *on the number and diameter of vessels in chorioallantoic membrane (CAM). Fertilized eggs were incubated at 37°C and 70% relative humidity in a forced draught incubator. The control eggs received sterile PBS only (a). At day 8, a window opening is punctured on each egg and 20 (b) or 40 *μ*g/egg (c) of the ethyl acetate fraction was injected into the chorioallantoic sac. 1, 2, and 3 are representative of heart, artery, and arterioles, respectively (magnification 10x). In the presence of 40 *μ*g/egg of ethyl acetate fraction, the diameter of vessels decreased significantly while the density of blood vessels did not change significantly ((d)–(f)). Data are mean ± SEM of three independent experiments performed in triplicate. *P < 0.05 versus untreated cells (0 *μ*g/egg).

To address whether* V. tricolor* inhibits angiogenesis, we examined the effect of EtOAc fraction of* V. tricolor *on CAM angiogenesis (Figures 6(a), 6(b), 6(c), 6(d), 6(e), and 6(f)). As shown in Figures 6(e) and 6(f), 40 *μ*g/egg of EtOAc fraction significantly decreased the diameter of vessels, while the number of newly formed blood vessels was not suppressed significantly, as compared to control CAM.

### 3.5. HPLC Profile of EtOAc Fraction of* V. tricolor*

**Figure 7:**
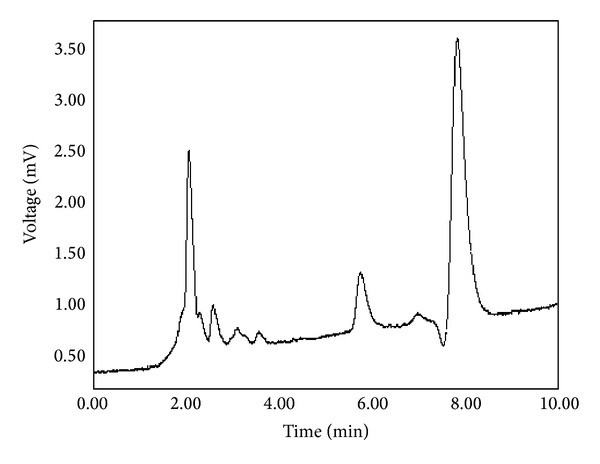
Representative HPLC chromatogram of ethyl acetate (EtOAc) fraction of* V. tricolor*. The peaks were monitored at 340 nm.

A simple and reliable HPLC fingerprint has been developed for qualification of the EtOAc fraction of* V. tricolor*. HPLC profile of EtOAc fraction under UV 340 nm was recorded. The corresponding HPLC chromatogram was presented in Figure 7. The fraction revealed 6 major peaks with retention time (RT) values in the range of 2.1 to 8.3 min for 20 *μ*L application volume (Figure 7).

## 4. Discussion

Heartsease (*Viola tricolor *L.) has a long history in treating inflammatory and skin disorders including scabs, itching, ulcers, eczema or psoriasis, and bronchitis or asthma [27, 28]. In the current work, we have studied the cytotoxic, apoptotic, and antiangiogenic activities of hydroalcoholic extract and EtOAc, *n*-butanol, and water fractions of* V. tricolor *on MCF-7, Neuro2a, and L929 cells. Cytotoxicity assay revealed that EtOAc fraction was the most potent among all the fractions with maximal effect on MCF-7 cells and minimal toxicity against normal cells. Apoptosis induction of EtOAc fraction (400 *μ*g/mL) was confirmed by increase in the sub-G1 peak of PI stained cells and western blot analysis of Bcl-2, Bax, and active form of caspase 3, as important proteins involved in the apoptotic cell death. Our results also showed that the diameter, but not the number, of blood vessels was significantly decreased by EtOAc fraction on CAM.

In this study, the* V. tricolor* hydroalcoholic extract was fractionated by solvent extraction with different polarity and the potential antitumor activity of low-polar solvent fraction (EtOAc) was compared to polar solvent fractions (*n*-BuOH and H_2_O). It was found that EtOAc fraction had the greatest antiproliferative activity in vitro. The effect of EtOAc fraction on nonmalignant cells showed a degree of specificity for malignant cell lines.

Pharmacognostic researches on* V. tricolor *confirmed the presence of high amount of saponins, mucilages, flavonoids, and phenolic compounds such as kaempferol, luteolin, quercetin, violanthin, and rutin, which placed* V. tricolor *in the list of plants with promising source of natural antioxidants [11, 12, 14]. In addition, bioactive plant cyclopeptides with intermediate polarity especially vitri A, vitri F, and cycloviolacin O2 are reported to have cytotoxic activity and are interesting candidates for drug development [29]. Phytochemicals with polyphenolic or flavonoid structure such as kaempferol, luteolin, and resveratrol have been reported to induce cancer cell death or to inhibit cancer cell proliferation by direct modulation of various molecular signal transduction pathways [30]. Because we used ethyl acetate fraction, it seems that intermediary polar constituents such as flavonoids and phenolic compounds are mainly involved in the cytotoxic activity of* V. tricolor*. The construction of chromatographic fingerprints plays an important role in the quality control of complex herbal medicines. Chemical fingerprints obtained by chromatographic techniques are strongly recommended for the purpose of quality control of herbal medicines, since they might represent appropriately the chemical integrities of the herbal medicines and therefore be used for authentication and identification of the herbal products [31]. In our chromatographic technique, in order to obtain a good resolution within a short analysis time, the composition of mobile phase was optimized. Various mobile phase compositions were evaluated. Acetonitrile and water containing phosphoric acid were chosen as the mobile phase because all peak components could be resolved under this condition. The acidification of mobile phase was beneficial, leading to good peaks separation and better peak shape. The HPLC fingerprint showed high stability and reproducibility and thus could be used for quality control of the EtOAc fraction and* Viola* products.

Current research on* V. tricolor *revealed new aspect of pharmacological properties of the herb. For example, bioactive cyclotides of* V. tricolor *has been demonstrated to possess inhibitory activity on proliferation of activated lymphocytes which may be beneficial in the therapy of disorders related to an overactive immune system [27]. Piana et al. showed the antinociceptive and anti-inflammatory activities of* V. tricolor *in the ultraviolet-B-induced skin burn [32]. There are also some researches which verified the anti-inflammatory activity of* V. tricolor *in traditional medicine [9, 33].

The crucial role of angiogenesis in tumor growth is well documented [34]. While new blood vessel formation on CAM was not suppressed by* V. tricolor*, a significant decrease in the diameter of vessels was seen. To our knowledge, it is the first time that an inhibition of angiogenesis on CAM by* V. tricolor* has been studied.

## 5. Conclusion 

Taken together, the result of present study showed that ethyl acetate fraction of* V. tricolor* has potential cytotoxic properties by decreasing proliferation of tumor cells, inducing apoptosis and inhibiting angiogenesis on CAM. These findings mean that further studies should be conducted in experimental animal models to evaluate the potential anticancer properties of* V. tricolor*.
